# Assessment of the impact of family physicians in the district health system of the Western Cape, South Africa

**DOI:** 10.4102/phcfm.v6i1.695

**Published:** 2014-12-17

**Authors:** Meyer Swanepoel, Bob Mash, Tracey Naledi

**Affiliations:** 1 Division of Family Medicine and Primary Care, Stellenbosch University, South Africa; 2 Health Programmes, Department of Health, Western Cape Government, South Africa

## Abstract

**Background**: In 2007, South Africa made family medicine a new speciality. Family physicians that have trained for this new speciality have been employed in the district health system since 2011. The aim of the present study was to explore the perceptions of district managers on the impact of family physicians on clinical processes, health system performance and health outcomes in the district health system (DHS) of the Western Cape.

**Methods:** Nine in-depth interviews were performed: seven with district managers and two with the chief directors of the metropolitan and rural DHS. Interviews were recorded, transcribed and analysed using the ATLAS-ti and the framework method.

**Results**: There was a positive impact on clinical processes for HIV/AIDS, TB, trauma, non-communicable chronic diseases, mental health, maternal and child health. Health system performance was positively impacted in terms of access, coordination, comprehensiveness and efficiency. An impact on health outcomes was anticipated. The impact was not uniform throughout the province due to different numbers of family physicians and different abilities to function optimally. There was also a perception that the positive impact attributed to family physicians was in the early stages of development. Unanticipated effects included concerns with their roles in management and training of students, as well as tensions with career medical officers.

**Conclusion**: Early feedback from district managers suggests that where family physicians are employed and able to function optimally, they are making a significant impact on health system performance and the quality of clinical processes. In the longer term, this is likely to impact on health outcomes.

**Evaluation de l'impact des médecins de famille dans le système de santé du district du Western Cape, en Afrique du Sud**.

**Contexte:** En 2007, l'Afrique du Sud a institué une nouvelle spécialité, la médecine de famille. Les médecins de famille qui se sont spécialisés dans cette nouvelle discipline sont employés dans le système de santé de district depuis 2011. L'objet de cette étude était d‘étudier les perceptions des gestionnaires de district sur l'impact que les des médecins de famille avaient sur les processus cliniques, la performance du système de santé et les résultats des systèmes de santé des districts (DHS) du Western Cape.

**Méthodes:** On a effectué neuf entrevues approfondies: sept avec les gestionnaires de district et deux avec les directeurs principaux du DHS rural et métropolitain. On a enregistré, transcrit et analysé les entrevues en utilisant ATLAS-ti et la méthode de structure.

**Résultats:** Il y a eu un effet positif sur les processus cliniques du VIH et/ou du SIDA, la Tuberculose, le traumatisme, les maladies chroniques non-contagieuses, la santé mentale, et la santé de la mère et de l'enfant. La performance du système de santé a été positivement affectée en termes d'accès, coordination, exhaustivité et efficacité. On s'attendait à un impact sur les résultats en matière de santé. L'impact n’était pas uniforme dans toute la province en raison du nombre différent de médecins de famille et des différentes capacités à fonctionner de manière optimale. On avait aussi l'impression que l'impact positif des médecins de famille en était aux premiers stades de développement. Les effets inattendus comprenaient leurs inquiétudes d'avoir à gérer et à former les étudiants, ainsi que les tensions avec les médecins de carrière.

**Conclusion:** Les premiers commentaires des directeurs de district indiquent que quand on emploie des médecins de famille qui ont la possibilité de fonctionner d'une manière optimale, ils ont un impact important sur la performance du système de santé et la qualité du processus clinique. Cela aura probablement un impact sur la santé, à long terme.

## Introduction

South Africa is following an international movement towards strengthening primary healthcare (PHC) services. This is being done in an attempt to improve the health of the citizens and reduce the cost of healthcare delivery.^1,2,3^ In South Africa, strategies for improving PHC and district health systems are being explored.^4,5^ The current national health insurance (NHI) roll-out has set the re-engineering of PHC as a priority.^6^

In 2007, the discipline of family medicine was recognised as a new speciality in South Africa, and as a result the eight South African medical schools changed their existing degree programmes to Master of Medicine (MMed). The Department of Health created full-time registrar training posts with family physician supervisors. The first Mmed-qualified family physicians graduated in 2011. In the Western Cape, 21 family physicians were employed in 2011, 32 in 2012 and 42 in 2014. It is expected that this number may increase to 60–80 over the next 5 years.

Nationally, there are 52 health districts, of which 6 are located in the Western Cape. The Cape Town metropolitan district is further sub-divided into 4 sub-structures. In the Western Cape, the Department of Health committed to establishing family physicians in a central role in the DHS, particularly at the district hospital, in order to improve patient outcomes. They do not provide first-contact care, but capacitate and support PHC teams in a clinical and mentorship capacity. They are custodians of clinical governance, provide leadership, and are catalysts in implementing community-orientated primary healthcare.^7,8^ The roles rest on the foundation of academic and clinical knowledge acquired within the new family medicine curriculum.

Overall, the perception is that the current family physicians in the Western Cape are making a difference, although no formal research has yet been conducted in a South African or African context.^7^ In the United States, Barbara Starfield showed that family physicians impact positively on health outcomes.^9^ Both Australia and the United Kingdom have established the central role of the trained family physician in the PHC system.^10^ In Brazil, the family healthcare teams, composed of one family physician, one nurse, one auxiliary nurse and 4–6 community health workers, serve 600–1000 families. They aim to provide first-contact care, as well as integrate care with acute level facilities.^11^ In Nepal, trained family physicians are making a significant difference to utilisation and provision of services at district hospitals.^12^

In the PHC re-engineering, apart from the roles described above, family physicians will also support community-based PHC teams. These teams will consist of community health workers and nurses, who will take responsibility for visiting a specific group of households. Family physicians will also form part of the district clinical specialist teams (DCSTs), which will, in addition, consist of an obstetrician, paediatrician, midwife, paediatric and PHC nurse. These specialist teams are meant to focus on improving maternal and child health services.^6^ In the Western Cape, such support is being offered in an alternative model by placing the family physicians at posts within the district and supporting them by outreach from the other specialists at the regional hospital. Therefore, no DCSTs, as envisaged by the national DoH, currently exist in the Western Cape. The new information from the present study, which is part of a larger study with the same title, will be useful to the Western Cape Department of Health in evaluating their strategy for the large-scale deployment of family physicians.^7^ This data will also be relevant in the broader South African context, considering the phased implementation of NHI.^6^ The Primafamed network (a network of departments of family medicine in Sub-Saharan Africa) will also be interested in the results of the research and the implications for their own countries.^5,13^ Sub-Saharan African policymakers may also find the results of the present study useful when uncertainty exists regarding their future commitment to the discipline of family medicine.

## Aim and objectives

The aim of the present study was to explore the perceptions of district managers on the impact of family physicians in the Western Cape. Specific objectives were to: (refer to Box 1 for greater detail):

BOX 1 **Definition of terms in study objectives.**

**Table:** 

Health system performance includes the following primary health care characteristics: **(14)**
Access (availability of services, geographic access, organisation of access, affordability, acceptability and satisfaction, utilization, equality of access).
Co-ordination (referrals, gate keeping, teamwork, skills-mix, integration of services between different levels and with community orientated primary care activities).
Comprehensiveness (range of equipment, range of first contact care, range of diagnoses dealt with, range of preventive services, range of procedures, range of promotive services).
Efficiency (Allocative or productive (e.g. waiting times, cost-effectiveness); technical (use of resources/laboratory tests); work force (referral rates, number consultations, duration of consultations, frequency of prescriptions).
Clinical processes refer to the care pathways and quality of care for key conditions across the burden of disease, and include: HIV, TB, child health, maternal health, trauma and emergency services, non-communicable diseases and mental health.
Health outcomes refer to measures of morbidity and mortality.

1. Explore the perceived effect of family physicians on health system performance.
2. Explore the perceived effect of family physicians on clinical processes.
3. Explore the perceived effect of family physicians on health outcomes.

## Methods

### Study design

This was a qualitative study using in-depth interviews.

### Setting

The present study was conducted in the public sector of the DHS of the Western Cape. The Western Cape DHS consists of four metropolitan sub-structures and five rural districts. Each of these entities has an overall manager, except the Central Karoo District, which is currently managed by the Eden district manager as the Eden/Central Karoo geographic service area. The four metropolitan sub-structures fall under a Chief Director of the Metro DHS and the five rural districts fall under a Chief Director of the Rural DHS. The Western Cape public health sector has aligned its Comprehensive Service Plan with the model of having a family physician at each district hospital (> 50 beds) and each community health centre (> 30 000 people served).

The researcher was a registrar in family medicine at the time of the research. Due to previous work as a clinical manager, he had an interest in health systems. He was interested to explore the effect of the appointment of well-positioned senior clinicians in the Western Cape DHS.

### Selection of participants

The managers of each of the rural districts and urban sub-structures in the Western Cape were selected for participation in the study, based on their broad scope of knowledge of the functioning of the DHS and overview of the impact that family physicians have made. In addition, the chief directors of the metropole (four sub-structures) and rural areas (five districts) were also selected.

### Data collection

In-depth interviews were performed face-to-face in the interviewee's office using an interview guide, and ranged from 60–80 mins. Interviews were performed in either English or Afrikaans by the researcher and were audiotaped. Interviews took the form of an open process of exploration that made use of open questions, reflective listening, summaries and clarification. The interview guide reflected the objectives of the study and ensured that all relevant points were explored. The audiotape of the first interview was reviewed by the first two authors to ensure that the guide was constructed appropriately and the interviewer demonstrated the necessary skills.

### Data analysis

Interviews performed in Afrikaans were translated into English prior to transcription and analysis. All interviews were transcribed and checked against the audiotapes by the researcher. Data were analysed using ATLAS-ti and the framework method. This involved the steps of: familiarisation with raw data; identifying an index of all the codes and categories to be used from the raw data; applying the index to all the raw data by annotating transcripts with the codes; charting all the raw data from the same code in a single document; and interpreting themes from the charts in terms of the range and strength of opinions, as well as any associations or relationships between themes.^15^ Quotations from these interviews are used in the present article to illustrate key points.

## Results

The study population consisted of nine interviewees, as described above. Three of them were male and 6 were female. All were middle aged (> 40 years) and had at least 5 years of experience in healthcare management. Two managers were very new in their positions, having served as facility managers for a number of years. One of the chief directors was also very new in the position, having served as a district manager for several years. One manager could not be reached for an interview; it is anticipated that this one interview would not have introduced new themes because no new issues emerged during the final interviews that were performed.

### General benefits

Respondents reported that overall family physicians had impacted the DHS positively, although benefits were perceived to be in the early stages of development. Those family physicians who impacted positively on the DHS earned the respect of colleagues. This respect was manifested in the number of applications received for family medicine training posts at these facilities:

‘You can actually see the places where family medicine is respected and where it's not by the number of applications we receive from the juniors.’

Graduates from the new training programme were considered to be well-trained and balanced clinicians, with a comprehensive skills set due to rotations through the 10 clinical domains. Senior registrars, during their third and fourth years of training, also benefitted the DHS in terms of their clinical impact:

‘You could already start seeing the impact that the registrars have in the senior years.’

### Roles of the family physician

The family physicians were perceived to have raised the clinical levels of functioning in the DHS, in terms of management of clinical emergencies, consultations on difficult cases and guidance to juniors to work more efficiently in terms of time and resources. They have taught juniors how to do ward rounds quicker and they have run specialist family physician clinics. Their seniority has given them the necessary authority to communicate directly with other consultants and senior managers:

‘You know it is just that seniority, that clinical knowledge, the more kind of decisiveness about what it is to be done.’

Clinical proficiency is the foundation for effective clinical governance, and family physicians were seen to be well suited for this function in the DHS, based on their comprehensive clinical training and the ensuing higher level of clinical reasoning. Regular morbidity and mortality meetings were held and recorded. Clinical audits to improve quality of care were done by means of folder reviews:

‘As a family physician she brings in the clinical governance oversight. So it's very much on clinical proficiency and whether you're making the right decisions.’

The role of family physicians appears to have evolved to that of a district family physician in some districts. Clinical governance in the sub-district still resided with the sub-district family physician, but one of these senior family physicians provided guidance to the sub-district family physicians in developing a clinical governance framework for their sub districts. The two senior family physicians had quarterly forums with the sub-district family physicians where they focused on clinical issues and lessons learned.

### Clinical processes

Family physicians were perceived to provide leadership for the campaign against diarrhoeal disease and helped co-ordinate activities. They identified the desired clinical outcomes and the appropriate course of action for each category of sick child in the diarrhoeal care pathway:

‘So there's a system that's put in place, but there's also that senior clinical decision making that's there and I think the diarrheal seasons is a great example of that.’

Respondents felt that the Integrated Management of Childhood Illness (IMCI) programme was impacted by family physicians, but the impact was limited. Those family physicians that had a clear understanding of the IMCI programme provided support and in-service training to nursing staff in order to attain better outcomes. Family physicians were perceived to impact the DHS positively in terms of mental health. One family physician was doing a situational analysis of the psychiatric services for the drainage area of their district hospital. He was working with the outreach psychiatrist and they were busy integrating the mental health services within the community, PHC facilities and the district hospital; as well as providing training and support for nursing staff. Some interviewees were of the opinion that the current DHS psychiatric care model is insufficient and it would be unfair towards the family physician to expect them to have a positive impact. However, some felt that even with the short comings of the current model that there are positive contributions that family physicians could make. Family physicians impacted positively on chronic disease management through clinical leadership and direct patient contact. They tended to think more holistically in terms of patient care. A particular family physician developed a risk stratification tool that was implemented in one district and used in the day-to-day care of non-communicable chronic diseases. This tool enabled trained healthcare providers to easily stratify these patients as either stable, at risk or decompensated according to appropriate clinical criteria applicable to each condition, and respond accordingly. These patients would then either continue with routine chronic disease care, or intensified chronic disease care, as characterised by more regular visits and special investigations, or by transfer to the casualty for possible admission to the hospital. This tool was later commissioned by the Western Cape Department of Health and piloted by several districts during their chronic disease drive. Many family physicians then implemented this risk stratification tool in their own sub-districts. They also impacted on chronic disease management by means of quality improvement cycles and staff training. Their inputs helped to integrate systems across a range of chronic diseases, rather than focusing on specific diseases:

‘I think the success is that we took a clinical governance perspective and not a programmatic perspective in implementing the chronic disease management plan. That to me it is really a strength and I think the family physician involvement there made a huge impact.’

Family physicians also had a significant impact on the care of patients with HIV and TB. They were seen as chipping away at the vertical nature of the HIV care model and providing a more holistic approach to patient care:

‘When she approaches chronic diseases she approaches it systemically. From where she's sitting that all chronic disease has got something in common and the systems that you put in place for good outcomes for the client is a uniform way of doing things.’

One family physician conducted a quarterly anti-retroviral (ARV) morbidity and mortality meeting and used this to capacitate nursing staff and doctors. She also communicated with the district office about routine ARV data, identifying the need for resources allocation and training gaps.

Maternal care in the metropolitan sub-structures was almost exclusively run by obstetricians, but in the rural districts maternal care was the domain of the generalist. Quality of clinical care at family physician managed facilities in the metropolitan areas was seen as on a par with other facilities of the metropolitan area. One family physician was tasked with improving prevention of mother-to-child transmission (PMTCT) rates. Good ante-natal care practices were implemented and PMTCT rates dropped:

‘So it's had a massive impact which I don't think we are capturing fully in that there's a story to be told about the impact of family medicine on the Midwife Obstetric Units, because I think it has been positive.’

It is recognised that the majority of patients with trauma and medical emergencies in the metropolitan area presented to the 24-hour community healthcare centres. The clinical governance and leadership that family physicians provided to medical officers in the emergency centres impacted indirectly on emergency care on a large-scale:

‘You know the family physicians take pride in the fact that they've actually with the full-time doctors, with the cosmos, with the general medical officers that work there – they have systematically improved the outcomes and the clinical decision making and the impact.’

### Health system performance

Respondents agreed that access to healthcare services was impacted positively by family physicians in different ways. This particularly included improved access to primary healthcare services via the clinical governance function of the family physician, which improved the quality and acceptability of care:

‘Look, I think access is probably the one area where family medicine has played a huge, huge impact. Because in our system ninety per cent of our contacts are on the primary health care platform.’

Access was also improved by expanding the clinical services offered by the DHS. This included the establishment of outreach services to new service points, as well as strengthening of existing outreach services from district hospitals to their feeding clinics by both qualified family physicians and registrars. This had the advantage of providing additional service delivery to the respective communities, whilst also training future family physicians within the context in which they will be expected to function in future. Consequently, a more seamless service and functional interface is created between the primary care services and the district level hospital. For example, one family physician analysed the referral and prescription patterns in the sub-district and introduced weekly outreach sessions by a family physician and nurse. This led to improved clinical care and adherence by the patients, and a reduction in emergency referrals.

Organisational access was positively impacted by family physicians. One family physician implemented a stratified approach to consultations, whereby the patient first consulted a clinical nurse practitioner (CNP). The consultation level was then escalated to the junior doctor, senior doctor and, eventually, the family physician as the need arose. This layered approach improved the quality of clinical decision making in their facility since there was a method of dealing with difficult cases:

‘When she got there three, four years ago, it was chaotic. The doctors there revolved and they didn't want to return to the place. So in four years she turned the place around.’

Patient satisfaction was affected directly by the intervention of family physicians. Respondents reported that family physicians were perceived by both staff and patients to be knowledgeable, senior and empathetic:

‘The child died at birth or just a few minutes after birth but the way in which the family physician and the staff supported that family was very positive. So it actually prevented the department from, you know from a legal case.’

Coordination of healthcare services in the district was also positively impacted by the family physician in many aspects:

‘I think the most important thing for me is really the person placing themselves at the centre of a team. And you know it's being at the centre of the team with a purpose. Being patient centred with a population outcome in mind.’

Family physicians impacted the referral system by means of appropriate improvements, such as training nursing staff at the clinic level to perform appropriate patient work-up prior to the referral. These family physicians were also perceived to be good at referring patients themselves as they know the referral routes and have more authority as a specialist. They were also perceived to have easier access to clinical management protocols. As the family physician in the sub-district they are more aware of all the problem cases and they also create awareness of referral problems at the geographic service area meetings where these issues may be solved. The range of procedures being done in the DHS was impacted significantly by family physicians. Training in surgical procedures was perceived to be appropriate and more of these procedures were being performed in the DHS. Equipment was being improved as a result:

‘I do think there's been a gradual introduction based on patient needs, of a wider range of services. And I think family physician has been at the centre of it.’

Family physicians were actively and routinely involved in curbing the cost of healthcare in the DHS. They considered the burden of disease profile and developed protocols for the cost-effective use of X-rays, laboratory services and medicines.

### Unanticipated effects

Family physicians were involved in meetings related to clinical governance but, in addition, they were unexpectedly drawn into more general district management meetings as well. This consumed the time of the family physician with management tasks they were not intended to perform:

‘What we didn't anticipate was that once we put family physicians on board everybody will call them into meetings’

Some family physicians also had joint appointments with the university, where they were responsible for certain academic responsibilities. It is perceived in some districts that the lack of description of standardised university and management tasks leads to a lessened impact as it removes the family physician from the DHS platform for a significant amount of time and becomes a source of misunderstanding and role uncertainty.

One district had no district manager and had also not appointed any family physicians, whilst it was reported that another had multiple district managers over a period of 4 years. This lack of consistent leadership may have undermined the potential contribution of family physicians who were working more like medical officers in this district. In another district, the introduction of the first family physicians created tension with the long-serving clinical managers and medical officers. The clinical managers were performing clinical governance functions and the medical officers at the district hospitals were perceived to be already sufficiently competent in their specific disciplinary areas. The need for family physicians was, therefore, not clear since the skills-mix they offered at the district hospital was already present and functioning well:

‘I think the fact that there are a lot of career medical officers and that there are medical managers together with the clinical managers, the family physicians are actually functioning as pure clinicians.’

Some districts commented on the financial implications of appointing family physicians. Creating a family physician post implied taking the budget away from a medical officer and nursing post, as there was no additional money. As the family physician does not just perform clinical care, this may be perceived as a loss of capacity at the coal-face.

### Outcomes

The general consensus was that family physicians definitely impact positively on health outcomes, although this perception needs further statistical verification:

‘Obviously that's anecdotal, so I would just say that my perception is, my indication is that it's probably better but that's only, that's only the impression.’

## Discussion

The findings suggest that family physicians are important contributors to improving the quality of primary and district hospital care. Refer to Box 2 for a summary of the health benefits of family physicians. The findings also underscore their value in contributing to the performance of the district healthcare system. Family physicians should, therefore, be important contributors to the implementation of local and national policy in the Western Cape. The findings resonate with the goals of the Healthcare 2030 policy in the Western Cape, which emphasises the need to build patient-centred quality of care and to improve district health services. The study findings also resonate with the goals of the national Negotiated Service Delivery Agreement, which emphasises the need to decrease maternal and child mortality rates, combat HIV/AIDS and TB as well as strengthen the health system.^16,17^

BOX 2 **Summary of key findings.**

**Table:** 

Key findings
An overall positive impact on health system performance and clinical processes.
Positive impact is in the early stages of development.
Positive impact is centred around individuals and not found uniformly throughout the district health services.
The expected roles of the family physician have been largely fulfilled.
FP have brought more clinical seniority into the district health services.

The findings are also congruent with the internationally recognised contribution of family physicians to strengthening health systems,^1^ and the resolution of the World Health Assembly on the importance of family physicians as part of a multi-disciplinary team in PHC (along with community health workers, primary health care nurses, midwives, and allied health professionals). The findings support the recommendations of the 2008 World Health Report that physicians with a specialisation in family medicine are needed to avoid a restrictive and watered-down approach to PHC in low-resource settings.^18^

Despite the mainly positive findings, the impact of these family physicians is still at an early stage and they have yet to fully evolve into their place within the developing DHS. In some areas, long-serving competent medical officers function as well as, and sometimes better than, newly qualified family physicians. Lower grade medical officer posts are not as expensive as family physicians and managers may wonder what the advantages are of employing family physicians. However, previous research has revealed significant clinical skills gaps in the district health services, due in part to variation in competencies between medical officers.^20^

Training programmes for family physicians aim to ensure a consistent set of competencies that are appropriate to the district health services, and an attractive career pathway for generalist doctors. In addition, family physicians are trained in a broader scope of practice that goes beyond clinical care to mentoring, capacity building, leadership, clinical governance and community-orientated primary care.^20^ In the future, it may be desirable for all doctors pursuing a long-term career in the district health services to be trained as family physicians. In the short to medium term, the picture will inevitably involve a mix of family physicians and medical officers working together to deliver the service.

The role of the family physician in terms of leadership, management and administration is variable, and respondents were worried that, as well-trained clinicians, they spent too much time in meetings. As a new speciality, the number of family physicians to support a wide range of management and planning processes in the sub-district and district is relatively small, which places a significant demand on each individual. The district health services expect the family physicians to make an impact on the continuum of care and clinical governance, which also requires their participation in a number of meetings.^16^ Most of the family physicians would be regarded as newly qualified and, usually, such junior consultants would focus more on clinical work and leadership within the immediate clinical team; however, the absence of senior colleagues forces many of them to take on significant leadership roles from the beginning – for example, being the first family physician in a newly opened district hospital. The intention is for family physicians to be expert generalists, working clinically, but with an important contribution to make to clinical leadership of the facility and DHS, rather than taking on the role of manager. More extensive management roles have been taken on by the few more-senior family physicians in the DHS.

Whilst there is an expectation that all family physicians would mentor and build capacity through training of the clinical teams where they work, some family physicians take on more-specific roles in relationship to teaching of undergraduate and postgraduate students.^7^ This teaching role and academic involvement at universities, at times, removed family physicians from the DHS platform (for example, teaching in the afternoon at the skills lab or involvement in clinical examinations). This remains a contentious issue between the universities and Department of Health and is currently the focus of new contractual agreements.^21^ These contracts will regulate access to the service platform for teaching, and also who will be regarded as joint staff with the university and have a more-specific academic role. Such joint staff would have job descriptions that indicate an academic component and would be allowed to attend specific training to capacitate them for this role.

In the 1990s, the introduction of training for doctors on the district health services platform was welcomed as a sign that medical schools were becoming more relevant, community-orientated and socially responsible. However, as the number of students and length of exposure has grown there is a valid concern that this is taking too much time away from service delivery and scarce resources, especially when this occasionally means teaching at the university campus. The district health services have no tradition of postgraduate training and the collaboration between service and academia, as exists in the tertiary hospitals. There is, thus, a need for both parties to better understand each other's perspectives and work towards developing a mutually beneficial culture of learning that leverages academic and service perspectives.^22^ Nevertheless, the need to increase the supply of doctors and family physicians is a national priority.^23^ Ultimately, the district health services are dependent on a supply of well and appropriately trained generalists, the universities are dependent on access to the district health services to provide this training, and both institutions must work together to fulfil their shared goal of improving health and the quality of health services.

### Limitations of the study methods

Senior healthcare managers do not work directly alongside family physicians and their perception might, thus, represent the opinion of others. Nevertheless, respondents were found to know family physicians by name and the impacts of individual family physicians were attributed specifically to individuals rather than generically. Respondents for the present study did not perform clinical work, and in some instances, respondents were not doctors, but had trained in another aspect of the health sciences such as nursing. Thus, their opinion on the impact of family physicians was not based on direct physical observation, but was a function of their managerial role and perspective within the DHS. They, therefore, represented an important overview of the district and were not necessarily biased towards a positive view of family physicians.

Interviewees may have been reluctant to express positive and negative perceptions about the impact of family physicians, due to the fact that the primary researcher was a family medicine registrar. This limitation was attenuated by explicitly exploring lack of impact or negative impact during the in-depth interviews.

A further limitation of this study was that it was set in the first year of appointment of newly graduated family physicians, with few other family physicians appointed in the DHS; their impact might not yet be fully developed.

### Implications or recommendations of the study for policy makers or future researchers

The proposed increase in the number of family physicians in the Western Cape DHS is supported by the present study's findings. It is anticipated that their positive impact will translate to improved outcomes and tangible improvements for staff and patients. The strengthening of the DHS through the deployment of family physicians should be considered by other South African provinces.

Family physicians that completed the new training curriculum were trained for the African context; however, the Western Cape is a different context to many African countries. Nevertheless, the findings of the present study could be transferable to other resource-constrained settings in Africa, and could add weight to the argument for the deployment of family physicians.

As part of a larger study, it is intended to interview managers on an annual basis for three years. This will allow researchers to qualitatively monitor changes in perceived impact over time. The findings from the present study can also be triangulated with findings from the larger study, which will provide quantitative evidence for the impact of family physicians.

## Conclusion

The appointment of family physicians in the DHS, who are trained within the new speciality training programmes, is in the early stages of development. They have impacted the DHS positively in terms of health system performance and clinical processes. It is anticipated that this will lead to a positive impact on health outcomes. The positive impact was centred on individual family physicians and was not found uniformly throughout the DHS. This refers to the fact that some districts did not have family physicians, and in other districts, family physicians were not able to function as intended due to a lack of other clinical staff. A few appeared to be under-performing because they had not yet grasped their leadership role and clinical governance function. The appointment and development of family physicians is a work in progress. The functioning of the DHS needs adjustment in some districts in order to better accommodate family physicians and enable them to have the impact that they are trained for.

## References

[1] De Maeseneer J, Haq C, Markuns J, et al The Contribution of Family Medicine to Improving Health Systems: A guidebook from the World Organization of Family Doctors. 2nd Ed Singapore: Wonca; 2013.

[2] Van Driel M, De Sutter A, Christiaens T, De Maeseneer J Quality of care: The need for medical, contextual and policy evidence in primary care. J Eval Clin Pract 2005;11(5):417–429. http://dx.doi.org/10.1111/j.1365-2753.2005.00549.x 16164582 10.1111/j.1365-2753.2005.00549.x

[3] Mash B, Blitz-Lindeque J Clinical skills for future family physicians. SA Fam Pract 2006;48(10):3 http://dx.doi.org/10.1080/20786204.2006.10873471

[4] Department of Health Healthcare 2020 - Western Cape Government [Homepage on the internet] c2011 [updated 2011, Nov; cited 2012, Feb 16]. Available from: http://www.westerncape.gov.za/other/2011/12/healthcare_2020_-_9_december_2020.pdf

[5] Chopra M, Lawn J, Sanders D, et al Health in South Africa 6: Achieving the Millenium Development goals for South Africa: challenges and priorities. The Lancet 374 9694 (Sep 19–Sep 25):1023–31. 10.1016/S0140-6736(09)61122-3 19709737

[6] Department of Health National health insurance: The first eighteen months. [Homepage on the internet] Pretoria; No date [cited 2013, Jun 8]. Available from: http://www.sarrahsouthafrica.org/LinkClick.aspx?fileticket=wgMmGElMkRk%3D&tabid=2311

[7] Nathan R, Rautenbach P Risks identified in implementation of district clinical specialist teams. South African Medical Journal, [S.l], 2013;103(3):144–146. 23472685 10.7196/samj.6551

[8] Mash B Reflections on the development of family medicine in the Western Cape, South Africa: A 15 year review. SA Fam Pract 2011;53(6):557–562. http://dx.doi.org/10.1080/20786204.2011.10874152

[9] Mash B, Reid S Statement of consensus on Family Medicine in Africa. Afr J Prm Health Care Fam Med. 2010;2(1),Art.#151, 4 pages.

[10] Macinko J, Starfield B, Shi L Quantifying the health benefits of primary care physician supply in the United States. International Journal of Health Services 2007;37(1):111–126. http://dx.doi.org/10.2190/3431-G6T7-37M8-P224 17436988 10.2190/3431-G6T7-37M8-P224

[11] Nicholson C, Jackson C, Marley J, Wells R The Australian Experiment: How Primary Health Care Organizations Supported the Evolution of a Primary Health Care System. JABFM 2012;25: Supplement S18–26. 10.3122/jabfm.2012.02.110219 22403246

[12] Paim J, Travassos C, Almeida C, Bahia L, Macinko J The Brazilian health system: History, advances, and challenges. The Lancet. 10.1016/S0140-6736(11)60054-8 21561655

[13] Nick Simons Institute [Homepage on the internet] http://www.nsi.edu.np.

[14] De Maeseneer J, Flinkenflögel M Primary health care in Africa: do family physicians fit in? Br J Fam Med 2010;60:286–292. 10.3399/bjgp10X483977 PMC2845490 20353673

[15] Kringos DS, Boerma WG, Hutchinson A, Van der Zee J, Groenewegen PP The breadth of primary care: a systematic literature review of its core dimensions. BMC health services research 2010;10:65 http://dx.doi.org/10.1186/1472-6963-10-65 20226084 10.1186/1472-6963-10-65 PMC2848652

[16] Pope C, Ziebland S, Mays N Qualitative research in health care: Analysing qualitative data. BMJ 2000;320:114–6. http://dx.doi.org/10.1136/bmj.320.7227.114 10625273 10.1136/bmj.320.7227.114 PMC1117368

[17] Department of Health Healthcare 2030. Cape Town: Western Cape Government; 2013 [cited 2014, Mar 28]. Available from: http://www.capegateway.gov.za/health

[18] Office of the Presidency Negotiated Service Delivery Agreement. For outcome 2: A Long and Healthy Life for All South Africans [Online] [cited 2014, Mar 28]; Available from: http://www.thepresidency.gov.za/MediaLib/Downloads/Home/Ministries/DepartmentofPerformanceMonitoringandEvaluation3/TheOutcomesApproach/Health%20Sector%20NSDA.pdf

[19] World Health Organisation World Health Report. Primary Health Care: Now More Than Ever [Homepage on the internet] c2014 [cited 2013, Jun 11]; Available from: http://www.who.int/whr/2008/en

[20] De Villiers MR, De Villiers PJT, Kent AP The maintenance of competence of rural district hospital medical practitioners. SA Family Practice 2006;48(3):18 http://dx.doi.org/10.1080/20786204.2006.10873353

[21] Couper I, Mash B, Smith S, Schweitzer B Outcomes for family medicine postgraduate training in South Africa. SA Fam Pract 2012;54(6):501–506. http://dx.doi.org/10.1080/20786204.2012.10874283

[22] Western Cape Government Western Cape Government Signs Multilateral Agreement for Health Professionals [Homepage on the internet] c2012 [upated 2012, May 28; cited 2014, Mar 31]. Available from: http://www.westerncape.gov.za/news/western-cape-government-signs-multilateral-agreement-health-professionals

[23] Jenkins L, Mash B, Derese A The national portfolio of learning for postgraduate family medicine training in South Africa: experiences of registrars and supervisors in clinical practice. BMC Medical Education 2013; 13:149 http://dx.doi.org/10.1186/1472-6920-13-149 24207009 10.1186/1472-6920-13-149 PMC4226197

[24] National Department of Health Human Resources for Health, South Africa. Pretoria: Department of Health; 2011.

